# Ending HIV transmission in Europe

**DOI:** 10.1016/j.lanepe.2025.101522

**Published:** 2025-11-07

**Authors:** Jan A.C. Hontelez, Casper Rokx

**Affiliations:** a Department of Public Health, Erasmus MC, University Medical Center Rotterdam, Rotterdam, the Netherlands; b Heidelberg Institute of Global Health (HIGH), Medical Faculty and University Hospital, Heidelberg University, Germany; c Department of Internal Medicine, Erasmus MC, University Medical Center Rotterdam, Rotterdam, the Netherlands; d Department of Medical Microbiology and Infectious Diseases, Erasmus MC, University Medical Center Rotterdam, Rotterdam, the Netherlands

Many European countries have managed to reduce HIV incidence tremendously over the past decades, yet declines have stalled, or even reversed, in many countries.^1^ By combining data from national HIV surveillance and population statistics, Jongen et al. provide a unique insight into the structural risk factors for HIV diagnosis in The Netherlands.^2^ HIV diagnoses were disproportionally found among individuals with a migrant background, and people with economic and mental health vulnerabilities. These data can help design tailored HIV treatment and prevention strategies to reach those at highest risk and ensure trends in incidence revert back to their downward trajectory.

HIV transmission control has two key components: protecting people without HIV through prevention interventions–such as pre-exposure prophylaxis (PrEP); and ensuring no onward transmission of people living with HIV through timely treatment initiation. Understanding who is at risk is essential to better target these interventions, and the study by Jongen et al.,^2^ among others, provides the data to do so. Nevertheless, some shortcomings should be considered. As noted by the authors, a key limitation is its cross-sectional design, with risk-factor assessment at the date of diagnosis. There can be many years between HIV acquisition and diagnosis, which hampers the causal inference of the identified factors. This is especially the case for mental health issues, which can emerge after HIV acquisition. Conversely, time-varying risk factors that may be associated with HIV acquisition may have lost their association, e.g. employment status or household composition. Furthermore, the association with age is likely somewhat confounded due to heterogeneities in time between diagnosis and infection: some of the higher risk identified at ages 25–49, as compared to <25, could be caused by delayed diagnoses rather than increased transmission. Additional analyses using existing approaches to estimate time of infection from surveillance data could remedy some of these shortcomings.^3^

The data from Jongen et al. point towards key vulnerable groups that may benefit from improved access to prevention, in particular migrants, women, and people with low income.^2^ In the Netherlands, PrEP access may vary with income, as PrEP pills currently need to be paid for out of pocket. Interventions such as the PREP2PEER initiative^4^—in which a “pay-what-you-can” model is used so that those with sufficient resources can pay for PrEP for those who cannot afford it themselves—may improve access among populations with low income. Furthermore, particularly for migrants, barriers to HIV prevention and testing services should be considered carefully,^5^ but more research is needed to understand to what extent HIV infection was already acquired pre-migration.

Timely treatment initiation is the second key component of effective HIV control, yet recent studies have demonstrated that a large proportion of HIV diagnoses in The Netherlands^6^ and Europe^7^ are at late stages of HIV. Risk factors for a new diagnosis, as reported by Jongen et al.,^2^ partly overlap with those for a late diagnosis (i.e. people with a migrant background and higher age), and most of these diagnoses occur in hospitals.^6^ This suggests either missed testing opportunities (general practitioners or medical specialists) or, as suggested by the authors, an inability to access HIV diagnostics earlier in the care process.

Testing based on HIV indicator conditions is an effective strategy to avoid missed opportunities for diagnosis in individuals who seek medical care for illnesses potentially linked to undiagnosed HIV. However, this approach remains underutilized across most European healthcare settings.^8^ The #aware.HIV project demonstrated that dedicated HIV teams, providing peer awareness, education, and feedback to physicians treating patients newly diagnosed with HIV indicator conditions, can significantly improve HIV testing rates (from 50.1% to 80.7%).^9^ However, accessing healthcare is a prerequisite, and poor PrEP uptake along with late HIV diagnoses may reflect deeper, systemic inequities in healthcare access. The pathway to timely HIV testing begins long before individuals present to a doctor with an indicator condition, and investing in education to enable a good start in life is a likely essential component for health access equity and timelier HIV testing.^10^

In conclusion, understanding the structural barriers to effective HIV prevention, testing, and treatment is essential to ensure that we cover the last mile towards ending HIV transmission in Europe. Emerging knowledge from research by Jongen et al. and others–and innovative projects such as PREP2PEER and #aware.HIV–are needed for the continued development of effective, tailored, and targeted interventions which improve access to HIV prevention and treatment services. At the same time, broader social-economic empowerment is also needed to ensure that people at risk can access these services.

## Contributors

JACH: writing-original draft; CR: writing-review & editing.

## Declaration of interests

Dr. Jan A.C. Hontelez has nothing to declare. Dr. Casper Rokx has received research grants from Gillead and ViiV health care, and has received consultation fees for scientific advisory committees at Gillead, ViiV health care, and Merck, Sharp & Dohme (MSD).
